# A Network Pharmacology Technique to Investigate the Synergistic Mechanisms of *Salvia miltiorrhiza* and *Radix puerariae* in Treatment of Cardio-Cerebral Vascular Diseases

**DOI:** 10.1155/2020/6937186

**Published:** 2020-10-05

**Authors:** Yang Ma, Wenjun Wang, Jiani Yang, Sha Zhang, Zhe Li, Fei Li, Shaojie Huang, Lu Lei, Kai Wang, Aidong Wen, Yi Ding

**Affiliations:** ^1^College of Pharmacy, Shaanxi University of Chinese Medicine, Xianyang, China; ^2^Department of Pharmacy, Xijing Hospital of the Fourth Military Medical University, Xi'an, China; ^3^Department of Basic Medicine, Shaanxi University of Chinese Medicine, Xianyang, China

## Abstract

**Objective:**

This study is aimed to analyze the active ingredients, drug targets, and related pathways in the combination of *Salvia miltiorrhiza* (SM) and *Radix puerariae* (RP) in the treatment of cardio-cerebral vascular diseases (CCVDs).

**Method:**

The ingredients and targets of SM and RP were obtained from Traditional Chinese Medicine Systems Pharmacology Database and Analysis Platform (TCMSP), and the disease targets were obtained from Therapeutic Target Database (TTD), National Center for Biotechnology Information (NCBI), and Online Mendelian Inheritance in Man (OMIM) Database. The synergistic mechanisms of the SM and RP were evaluated by gene ontology (GO) enrichment analyses and Kyoto encyclopedia of genes and genomes (KEGG) path enrichment analyses.

**Result:**

A total of 61 active ingredients and 58 common targets were identified in this study. KEGG pathway enrichment analysis results showed that SM- and RP-regulated pathways were mainly inflammatory processes, immunosuppression, and cardiovascular systems. The component-target-pathway network indicated that SM and RP exert a synergistic mechanism for CCVDs through PTGS2 target in PI3k-Akt, TNF, and Jak-STAT signaling pathways.

**Conclusion:**

In summary, this study clarified the synergistic mechanisms of SM and RP, which can provide a better understanding of effect in the treatment of CCVDs.

## 1. Introduction

Cardio-cerebral vascular diseases (CCVDs) seriously threaten human health and life for the high occurrence and mortality [1]. Severe sequelae, complicated symptoms, and difficulties in treatment are markedly characteristics of CCVDs. CCVDs include cerebrovascular diseases and cardiovascular diseases, and about 40% of middle-aged and elderly people are suffering from hyperglycemia, hyperlipidemia, and hypertension [2]. Therefore, it is of great clinical significance to formulate effective treatment strategies for the significant characteristics of CCVD such as severe sequelae, complex symptoms, and difficult treatment [3].

*Salvia miltiorrhiza* (SM), a Traditional Chinese Medicine (TCM), has been widely used in the treatment of CCVDs [4]. *Radix puerariae* (RP) is one of the earliest medicinal materials used in ancient China, and the effects on CCVDs in the elderly have been reported [5]. The combination of SM and RP exerts synergistic effect on CCVDs. Studies have shown that SM and RP are well tolerated in the treatment of cardio-cerebral vascular diseases and can improve neointimal hyperplasia, vascular function, and structure and significantly improve the occurrence of atherosclerosis and hypertension [6, 7].

SM has been extensively used in Asian countries against CCVDs. The primary active ingredients of SM are water-soluble phenolic acids represented by salvianolic acid B and fat-soluble ketones represented by tanshinone IIA. In previous studies, it has been proved that SM has improved microcirculation, inhibition of platelet aggregation, and antioxidation [8, 9]. RP also showed significant effects on senile cerebrovascular disease, cardiovascular disease, and neurodegenerative diseases [10, 11]. SM and RP were applied in blood stasis, and RP promoted vascular smooth muscle movement and blood circulation. SM can remove blood stasis and blood slow flow caused by blood stasis to ensure blood flow unimpeded. SM and RP can synergistically affect CCVDs [12]. Lam et al. [13] have demonstrated SM and RP can potentially improve cerebrovascular circulation. However, the mechanism of action of SM and RP is not clear.

**Figure 1:**
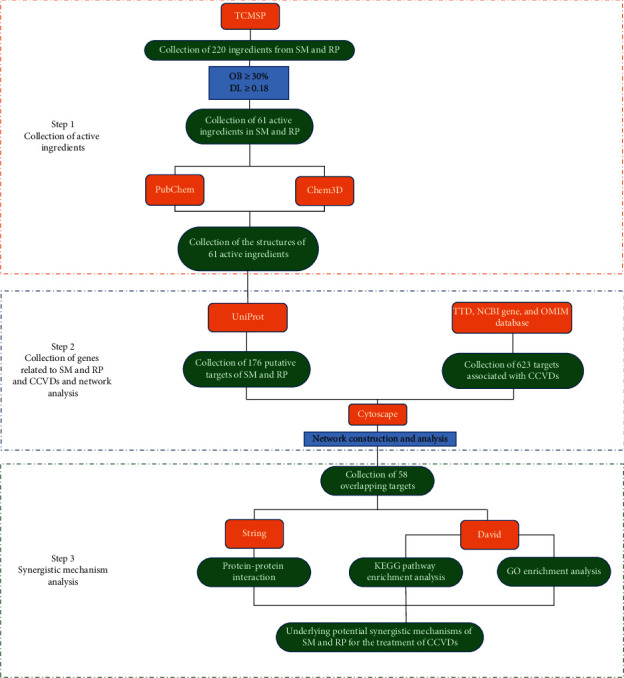
Workflow of network pharmacology analysis.

The network pharmacology approaches, concentrated on analyzing the network connectivity and dynamics as ingredients of drug targets and designing the optimal therapeutic strategies, can expose the underlying complex relationships between the herbal formula and the whole body [14]. Coincidentally, almost all Chinese medicines and ethnic medicine around the world work by targeting multiple molecules on the human body [15]. It takes advantage of advancements in systems biology, a high degree of integration data analysis strategy, and interpretable visualization provides deeper insights into the underlying mechanisms of TCM theories, including the principles of herb combination, biological foundations of herb or herbal formulae action, and molecular basis of TCM syndromes [16]. In this study, the mechanism of SM and RP on CCVDs was studied by the network pharmacology method, which provided a theoretical basis for the further research of SM and RP.  Figure 1 is the network pharmacology analysis workflow.

## 2. Materials and Methods

### 2.1. Active Ingredients of SM and RP

It is useful to explore the molecular mechanisms based on pharmacokinetics characteristics [17]. SM and RP are mostly used by oral administration in the clinic; therefore, pharmacokinetics parameters such as oral bioavailability (OB) and drug-likeness (DL) were investigated. OB is commonly used to measure whether oral drugs can be through obstacles as well as be transported into the systemic blood circulation. DL is mainly used to predict exactly how “drug like” an ingredient is, which helps to assist pharmacokinetic and pharmaceutical properties, for example, solubility and chemical stability [18]. The active ingredients of SM and RP were gathered from TCMSP [19] (https://tcmspw.com/tcmsp.php), a phytochemical database, with the keywords “*Salvia miltiorrhiza*” and “*Radix puerariae*”. The OB and DL of chemical ingredients of SM and RP were collected from TCMSP. In this study, screening standard was set to OB ≥ 30% and DL ≥ 0.18, and chemical ingredients lower than this standard were eliminated from the final list of active ingredients of SM and RP.

### 2.2. Ingredient Targets of SM and RP

All proteins related to the active ingredients were obtained from the TCMSP databases. Protein names of SM- and RP-related protein were unified to official target name by UniProt [20] (https://www.uniprot.org/), with limitation to “*Homo sapiens.*” Subsequently, protein names were transformed into official target name and duplicates were deleted.

### 2.3. Targets of CCVDs

Disease association targets were acquired from the TTD [21] (http://bidd.nus.edu.sg/group/cjttd/), NCBI [22] (https://www.ncbi.nlm.nih.gov/gene) and the OMIM [23] (http://www.omim.org/) database, and merely, “*Homo sapiens*” proteins connected to CCVDs were chosen. UniProt was used to unify the name of target associated with CCVDs because the target information of the disease is derived from different databases and has a nonstandardized name, and the duplicates were deleted. Active ingredients and disease targets were uploaded into the Veeny 2.1 (http://bioinfogp.cnb.csic.es/tools/venny/) website to obtain common targets of active ingredients and disease.

### 2.4. GO and KEGG Enrichment Analysis

Common targets of ingredients and disease were uploaded to Visualization and Integrated Discovery (David) [24] variation 6.8 (https://david.ncifcrf.gov/), and the species was set as “*Homo sapiens*” for GO and KEGG enrichment analysis. Among them, GO enrichment analysis includes three parts: cellular component (CC), biological process (BP), and molecular function (MF). A histogram was drawn according to the analysis results.

### 2.5. Common Target PPI Network Construction

The protein-protein interaction (PPI) information was obtained from the Search Tool for the Retrieval of Interacting Genes [25] (STRING, https://string-db.org/) database by uploading the common targets with the species limited as “*Homo sapiens*” (human) and “minimum required interaction score: highest confidents (0.900)”. Other parameters remain the default settings, and the interactive relationship will be obtained. PPI network was drawn by importing the interaction information in Cytoscape 3.6.1; thereafter, topological parameter was examined by using the network analyzer in the software.

### 2.6. Construction of Active Ingredient-Target-Pathway Network

The targets involved in the main pathways were listed, and duplicates were removed and ligated with the active ingredients of SM and RP. The active ingredient-target-pathway network was constructed with Cytoscape 3.6.1 software.

## 3. Results

### 3.1. Active Ingredients and Target Acquisition of SM and RP

**Figure 2:**
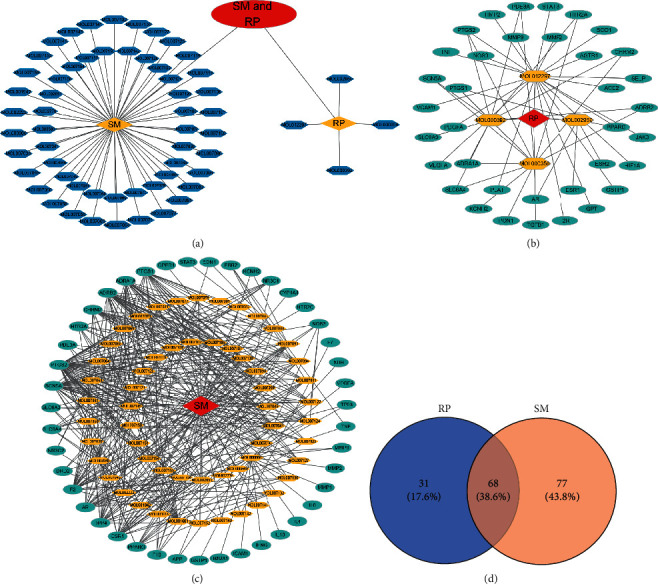
Linkage of target compounds and target genes. (a) The network of herbal medicine compound in SM and RP. The yellow diamond is SM and RP, and the blue pentagon is the active ingredient. (b, c) The component-target diagram of SM and RP. The yellow hexagon is the active ingredients, and the green ellipse is the target. (d) The Veeny diagram of the target genes for SM and RP.

In SM and RP, 61 active ingredients and 176 targets were obtained from TCMSP. The resulting compound-target network is shown in Figures 2(a)–2(c), respectively.  Figure 2(d) shows that there are 68 common targets in SM and RP. The active ingredients information about SM and RP is shown in Table 1. The structural formula was drawn with ChemDraw software. Active ingredients information is presented in Supplementary Table S1. The structure of active ingredients data is provided in Supplementary Document 1.

### 3.2. CCVDs Target Acquisition

The targets of disease gained from the TTD, NCBI, and OMIM databases. These targets were uploaded into the UniProt database for correction, and 623 targets were included after the deletion of the duplicates. The targets of the active ingredients and disease were used to acquire the common target through the Veeny 2.1 website. These targets are both targets of drug and disease. Therefore, SM and RP are likely to play a salutary role through these targets. Disease target information is provided in Supplementary Table S2. Disease-drug common targets data are provided in Supplementary Table S3.

### 3.3. GO Enrichment Analysis

**Figure 3:**
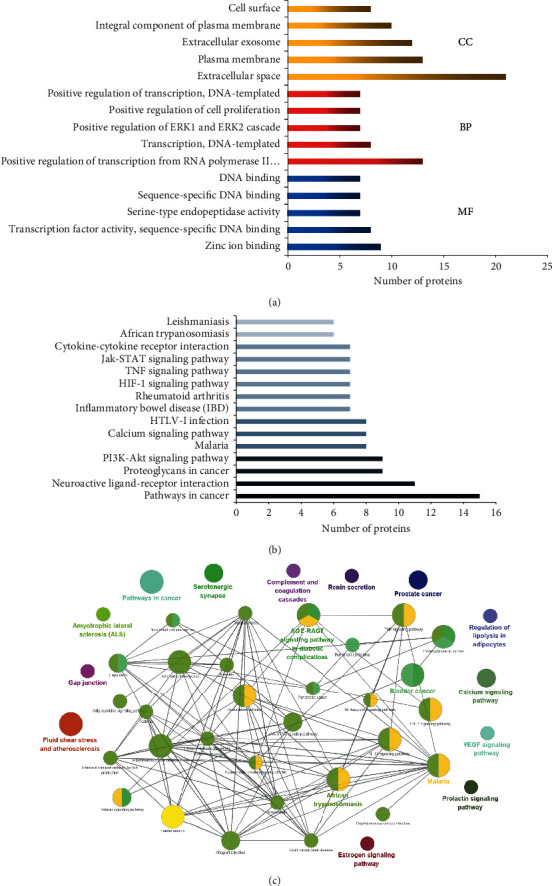
KEGG pathways and GO analysis by David database: (a) GO analysis of candidate targets. Database showed the five remarkably enriched items in the biological processes (BP), cell component (CC), and molecular function (MF); (b) KEGG pathways of target genes; (c) main functional annotation clusters by Biocarta analysis.

GO enrichment analysis result was obtained from the David database. The top five count value was selected (Table 2) to draw the histogram (Figure 3(a)). The results showed that, with the enrichment results of BP, the target of SM and RP treatment of CCVDs was generally associated with the policy of RNA polymerase II promoter transcription, DNA template transcription, and cell proliferation. According to the enrichment consequence of CC, the target of SM and RP in treating CCVDs was mainly focused on the extracellular space, plasma membrane, and extracellular exosomes. From the results of MF enrichment, the effect of SM and RP on the treatment of CCVDs is mainly zinc ion binding, serine endopeptidase activity, and sequence-specific DNA binding. GO analysis data are provided in Supplementary Table S4 (sheet1, sheet2, and sheet3).

### 3.4. KEGG Pathway Enrichment Analyses

The KEGG analysis results from the David database; the top 15 count value was selected and the specific data, as shown in Table 3. A histogram was drawn, and the results are presented in Figure 3(b). The KEGG analysis results were imported in the Cytoscape software. The results are shown in Figure 3(c). It can display in table that it includes inflammatory processes and immunosuppression (such as TNF signaling pathway), cardiovascular systems (such as PI3K-Akt and JaK-STAT signaling pathway) and neuroactive ligand-receptor interaction. The principal processes of SM and RP in the treatment of CCVDs consist of signaling paths such as PI3K-Akt, TNF, and JaK-STAT signaling pathway. KEGG pathway analysis data are provided in Supplementary Table S4 (sheet4).

### 3.5. Component-Target-Pathway Network Analyses

**Figure 4:**
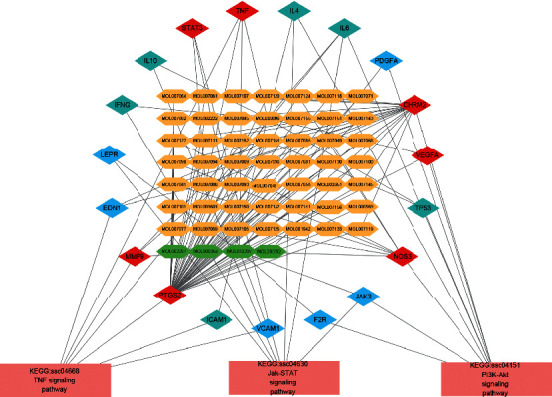
Component-target-path network. The blue diamond represents the target, the red diamond represents the cotarget, the yellow hexagon represents the SM active component, the green hexagon represents the RP active component, and the pink rectangle represents the pathway.

The targets involved in PI3K-Akt, TNF, and JaK-STAT signaling pathway were listed and removed the duplicates, and the results were as follows: IL4, IL6, PDGFA, CHRM2, VEGFA, TP53, NOS3, JAK3, F2R, VCAM1, ICAM1, TNF, PTGS2, MMP9, EDN1, LEPR, IFNG, IL10, and STAT3, a total of 19 targets. Then, find these targets and refer to the ingredients using Cytoscape 3.6.1 software to construct an ingredient-target-pathway network, as shown in Figure 4.

Figure 4 shows the ingredient-target-pathway network. The blue diamond represents the target, the red diamond represents the cotarget, the yellow hexagon represents the SM active component, the green hexagon represents the RP active component, and the pink rectangle represents the pathway.

### 3.6. Common Target PPI Network Constructions

**Figure 5:**
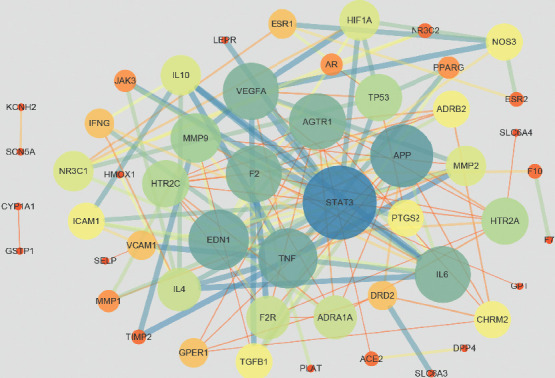
Protein-protein interaction (PPI) networks of active ingredients of SM and RP for the treatment of CCVDs. Each node represents the relevant gene, and the edge means line thickness indicates the strength of data support. PPI network map of active components and diseases.

The interactive relationship of protein-protein was obtained through the STRING database, and then the target protein PPI network map was drawn by Cytoscape 3.6.1, as shown in Figure 5. The network consists of 58 nodes with 130 edges, an average degree of 4.48, and a PPI enrichment *p* value: <1.0e−16. In the figure, the greater the degree, the bigger the node.

## 4. Discussion

TCM usually plays a multi-ingredient and multipathway synergistic effect against various diseases. Many research studies have shown that network pharmacology has made great improvement in exploring the application of active ingredients, targets, and systems in TCM [26, 27]. Therefore, this research method applied to TCM should correspond to the synergistic mechanism. In this study, we applied a network pharmacology approach to investigate the related targets and pathways of the combination of SM and RP against CCVDs, thereby illuminating the synergistic mechanism of SM and RP on CCVDs.

In this study, the network pharmacology method was conducted to explore the synergistic effect of SM and RP on CCVDs to enhance the accuracy of target forecast to some extent. We found 61 active ingredients in SM and RP and 58 targets related to CCVDs. Based on the outcomes of pathway enrichment, SM and RP active ingredients can simultaneously target cancer pathways, neuroactive ligand-receptor interactions, and PI3K-Akt and TNF signaling pathways, leading to the synergistic effect of SM and RP. Furthermore, depending on the PPI system analysis, STAT3, APP, EDN1, TNF, AGTR1, VEGFA, IL6, F2, MMP9, and HTR2C were recognized as the center targets. These targets are synergistic targets of SM and RP treatment of CCVDs.

In the PPI system analysis, STAT3, APP, EDN1, TNF, AGTR1, VEGFA, IL6, F2, MMP9, and HTR2C were considered to be crucial targets. TNF, a cytokine related to a severe stage of systemic inflammation, is substantially raised in patients with ischemic and hemorrhagic stroke [28]. Besides, VEGFA is a member of the VEGF family of proteins [29]. VEGFA has proangiogenic and neuroprotective effects that induce neurogenesis [30] and is increasingly important in the systemic treatment of CCVDs [31]. IL6 is a cytokine originated from T cells and macrophages that has been involved in many types of biological activities such as the formation the severe phase responses and regulation of the organic immune system, and it is related to the metabolic processes during exercise [32]. Primary studies showed that IL6 is the best proinflammatory biomarker in case of stroke [33]. IL6 can also be activated by immune effect damaged by ischemia reperfusion (I/R) [34]. MMPs are well-known mediators of cardiovascular pathophysiology. MMP9 is an important mediator of cardiac remodeling after MI and is centrally involved in inflammation and repair components of the response [35].

GO and KEGG analysis was performed to better understand the interaction of targets. In the result, GO analysis exposed that the target is mainly involved in transcriptional favorable policy of RNA polymerase II promoter, transcription, DNA template, positive policy of ERK1 and ERK2 cascades, and biological processes of and biological processes of negative policy of cell proliferation; the molecular practical body is primarily managed by zinc ion binding, transcription factor activity, serine endopeptidase activity, sequence-specific DNA binding, and so forth. Cell partial analysis revealed that the extracellular area represented the largest proportion, followed by the plasma membrane, extracellular exosomes, the integral components of the plasma membrane, and the cell surface. Interestingly, KEGG pathway analysis is mostly involved in neuroactive ligand-receptor interactions, PI3K-Akt, TNF, and Jak-STAT signaling pathways, which are consistent with previous reports that are involved in the progression of key features in CCVDs.

The phosphatidylinositol 3-kinase (PI3K-Akt) pathway plays an essential role in intracellular signal transduction involved in cell proliferation, cell survival, inflammation, and metabolism [36]. Neuronal apoptosis is the main performance of cell death following cerebral ischemia. One of the important cellular mechanisms, the balance between apoptosis and antiapoptotic signals, figures out the fate of nerve cells after cerebral I/R [37]. There is evidence showed that the PI3K-Akt pathway can be activated to regulate cell apoptosis and cerebral I/R injury, thereby playing a significant neuroprotective effect [38, 39]. Studies have shown that inhibiting AKT phosphorylation attenuates neuronal apoptosis against cerebral I/R injury [40, 41]. Studies have shown puerarin and tanshinone IIA synergistic effect on the PI3K-Akt pathway to protect CCVDs [42, 43]. TNF signaling pathway can promote the expression of proinflammatory cytokines, chemokines, growth factors, and TNF-*α* itself to amplify the inflammatory response and immune effects [44]; Janus kinases/signal transducer and activator of transcription (Jak-STAT) signaling pathway is a crucial signaling pathway in cells, which is involved in mediating cardiomyocyte growth, development, apoptosis, and regulation of angiogenesis. Cagnin et al. [45] show that microarray and meta-analysis of human coronary atherosclerotic plaque modification gene expression discovered that the Jak-STAT pathway plays an important role in cardiovascular protection. As shown in Figure 4, the highest value of the degree of the target interacting with the ingredients is PTGS2. In addition, PTGS2, called cyclooxygenase 2, is an important enzyme in the biosynthesis of prostaglandins, which has both a dioxygenase and a peroxidase [46]. Thus, SM and RP synergistically effect with PI3K-Akt, TNF, and Jak-STAT signaling pathways and PTGS2 targets to treat CCVDs.

In summary, this study explored the related diseases and complex diseases from the perspective of systemic pharmacology to identify active ingredients and improve cognition of the effective mechanism of TCM. We mainly from the following aspects to study the synergistic effects of SM and RP on CCVDs, such as the active ingredients in SM and RP, related targets, signal pathways, and biological processes involved in the related targets. In general, all nodes in the PPI network directly or indirectly affect the pathological process of CCVDs. Through the ingredient-target-pathway network, it was found that SM and RP play a synergistic therapeutic role mainly through PTGS2 target and PIK3-Akt, HIF-1, Jak-STAT, and TNF signaling pathways. This study first theoretically explained the synergistic therapeutic effect of SM and RP on CCVDs and further explained the principles of compatibility of TCMSP. On the other hand, it explored the active ingredients, targets, and pathways of SM and RP in the treatment of CCVDs to illustrate the synergistic effect, and it also provided reference for further study of pharmacology experiment of CCVDs treatment and other related studies of TCM.

## Figures and Tables

**Table 1:** Table of main active ingredients of SM and RP.

Mol id	Components	Structure	OB (％)	DL (％)
SM MOL001601	1,2,5,6-Tetrahydrotanshinone	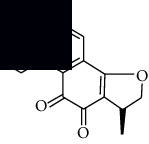	38.75	0.36
MOL001659	Poriferasterol	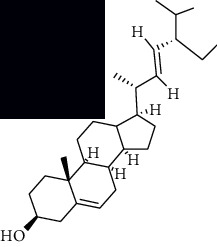	43.83	0.76
MOL001942	Isoimperatorin	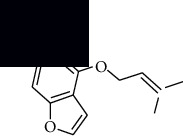	45.46	0.23
MOL002222	Sugiol	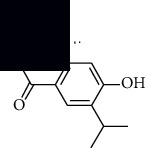	36.11	0.28
MOL002651	Dehydrotanshinone II A	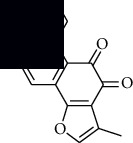	43.76	0.4
MOL002776	Baicalin	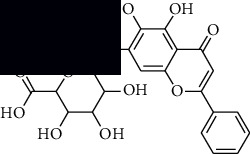	40.12	0.75
MOL000569	Digallate	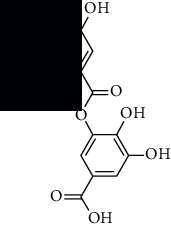	61.85	0.26
MOL000006	Luteolin	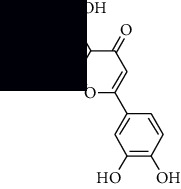	36.16	0.25
MOL007036	5,6-Dihydroxy-7-isopropyl-1,1-dimethyl-2,3-dihydrophenanthren-4-one	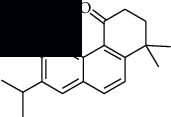	33.77	0.29
MOL007041	2-Isopropyl-8-methylphenanthrene-3,4-dione	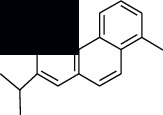	40.86	0.23
MOL007045	3*α*-HydroxytanshinoneIIa	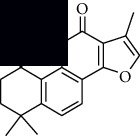	44.93	0.44
MOL007048	(E)-3-[2-(3, 4-Dihydroxyphenyl)-7-hydroxy-benzofuran-4-yl]acrylic acid	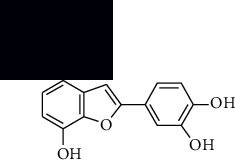	48.24	0.31
MOL007049	4-Methylenemiltirone	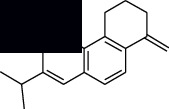	34.35	0.23
MOL007050	2-(4-Hydroxy-3-methoxyphenyl)-5-(3-hydroxypropyl)-7-methoxy-3-benzofurancarboxaldehyde	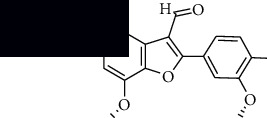	62.78	0.4
MOL007058	Formyltanshinone	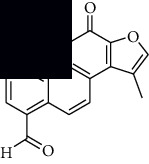	73.44	0.42
MOL007059	3-*β*-Hydroxymethyllenetanshiquinone	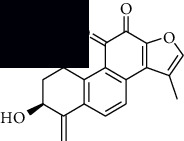	32.16	0.41
MOL007061	Methylenetanshinquinone	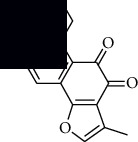	37.07	0.36
MOL007063	Przewalskin a	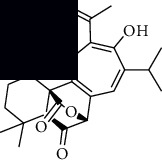	37.11	0.65
MOL007064	Przewalskin b	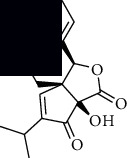	110.32	0.44
MOL007068	Przewaquinone B	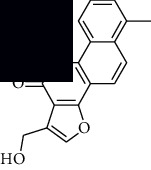	62.24	0.41
MOL007069	Przewaquinone c	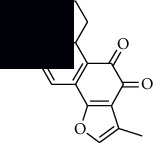	55.74	0.4
MOL007070	(6S, 7R)-6,7-Dihydroxy-1,6-dimethyl-8,9-dihydro-7H-naphtho[8,7-g]benzofuran-10,11-dione	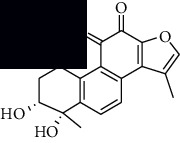	41.31	0.45
MOL007071	Przewaquinone f	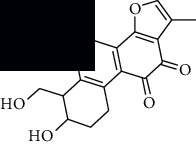	40.31	0.46
MOL007077	Sclareol	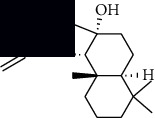	43.67	0.21
MOL007079	Tanshinaldehyde	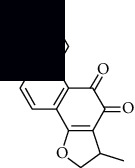	52.47	0.45
MOL007081	Danshenol B	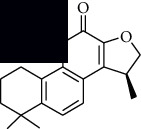	57.95	0.56
MOL007082	Danshenol A	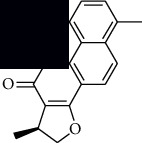	56.97	0.52
MOL007085	Salvilenone	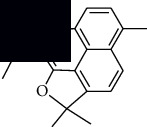	30.38	0.38
MOL007088	Cryptotanshinone	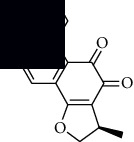	52.34	0.4
MOL007093	Danshexinkum d	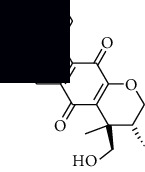	38.88	0.55
MOL007094	Danshenspiroketallactone	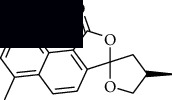	50.43	0.31
MOL007098	Deoxyneocryptotanshinone	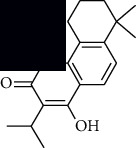	49.4	0.29
MOL007100	Dihydrotanshinlactone	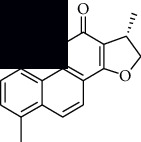	38.68	0.32
MOL007101	DihydrotanshinoneI	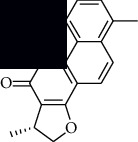	45.04	0.36
MOL007105	Epidanshenspiroketallactone	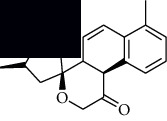	68.27	0.31
MOL007107	C09092	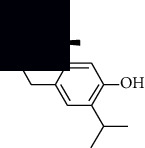	36.07	0.25
MOL007108	Isocryptotanshi-none	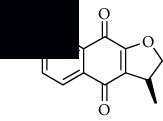	54.98	0.39
MOL007111	Isotanshinone II	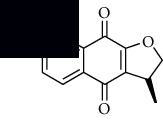	49.92	0.4
MOL007119	Miltionone I	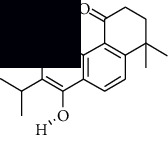	49.68	0.32
MOL007120	Miltionone II	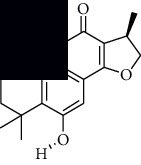	71.03	0.44
MOL007121	Miltipolone	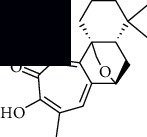	36.56	0.37
MOL007122	Miltirone	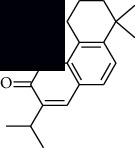	38.76	0.25
MOL007124	Neocryptotanshinone ii	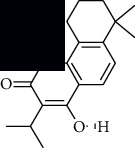	39.46	0.23
MOL007125	Neocryptotanshinone	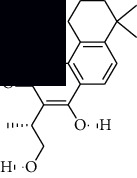	52.49	0.32
MOL007127	1-Methyl-8,9-dihydro-7H-naphtho[5,6-g]benzofuran-6,10,11-trione	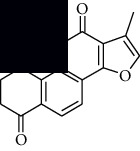	34.72	0.37
MOL007130	Prolithospermic acid	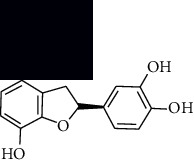	64.37	0.31
MOL007132	(2R)-3-(3,4-Dihydroxyphenyl)-2-[(Z)-3-(3,4-dihydroxyphenyl)acryloyl]oxy-propionic acid	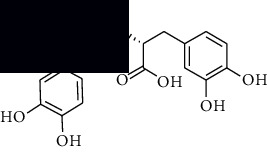	109.38	0.35
MOL007141	Salvianolic acid g	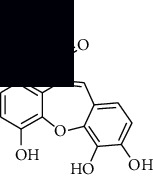	45.56	0.61
MOL007142	Salvianolic acid j	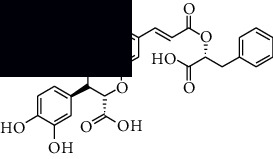	43.38	0.72
MOL007143	Salvilenone I	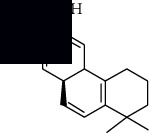	32.43	0.23
MOL007145	Salviolone	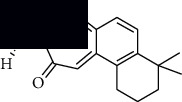	31.72	0.24
MOL007150	(6S)-6-Hydroxy-1-methyl-6-methylol-8,9-dihydro-7H-naphtho[8,7-g]benzofuran-10,11-quinone	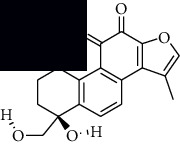	75.39	0.46
MOL007151	Tanshindiol B	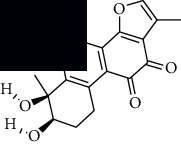	42.67	0.45
MOL007152	Przewaquinone E	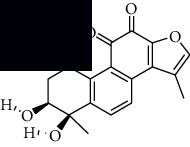	42.85	0.45
MOL007154	Tanshinone iia	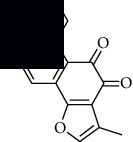	49.89	0.4
MOL007155	(6S)-6-(Hydroxymethyl)-1,6-dimethyl-8,9-dihydro-7H-naphtho[8,7-g]benzofuran-10,11-dione	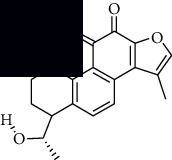	65.26	0.45
MOL007156	Tanshinone ?	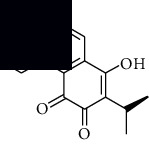	45.64	0.3
RP MOL000392	Formononetin	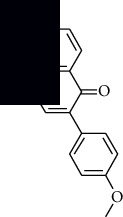	69.67	0.21
MOL000358	Beta-sitosterol	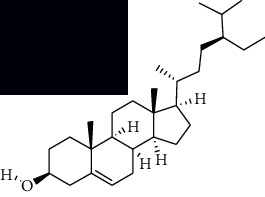	36.91	0.75
MOL002959	3'-Methoxydaidzein	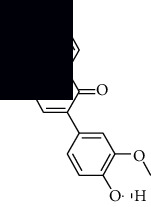	48.57	0.24
MOL012297	Puerarin [5]	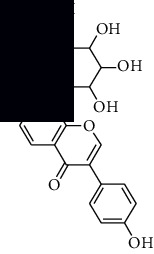	24.03	0.39

**Table 2:** GO enrichment analysis results.

	GO enrichment	Count
MF	Zinc ion binding	9
	Transcription factor activity and sequence-specific DNA binding	8
	Serine-type endopeptidase activity	7
	Sequence-specific DNA binding	7
	DNA binding	7
CC	Extracellular space	21
	Plasma membrane	13
	Extracellular exosome	12
	Integral component of plasma membrane	10
	Cell surface	8
BP	Positive regulation of transcription from RNA polymerase II promoter	13
	Transcription, DNA templated	8
	Positive regulation of ERK1 and ERK2 cascades	7
	Positive regulation of cell proliferation	7
	Positive regulation of transcription, DNA-templated	7

**Table 3:** KEGG pathway enrichment analysis.

KEGG pathway	Number of targets	Count
Pathways in cancer	IL6, AR, PTGS2, PDGFA, MMP9, PPARG, TP53, MMP2, TGFB1, MMP1, STAT3, AGTR1, HIF1A, VEGFA, F2R	15
Neuroactive ligand-receptor interaction	AGTR1, ADRB2, DRD2, CHRM2, LEPR, F2, ADRA1A, NR3C1, HTR2C, F2R, HTR2A	11
Proteoglycans in cancer	HIF1A, TNF, MMP9, VEGFA, ESR1, TP53, MMP2, TGFB1, STAT3	9
PI3K-Akt signaling pathway	IL4, IL6, PDGFA, CHRM2, VEGFA, TP53, NOS3, JAK3, F2R	9
Malaria	VCAM1, ICAM1, SELP, IL6, TNF, IFNG, TGFB1, IL10	8
Calcium signaling pathway	AGTR1, ADRB2, CHRM2, ADRA1A, NOS3, HTR2C, F2R, HTR2A	8
HTLV-I infection	VCAM1, ICAM1, IL6, TNF, PDGFA, TP53, JAK3, TGFB1	8
Inflammatory bowel disease (IBD)	IL4, IL6, TNF, IFNG, TGFB1, IL10, STAT3	7
Rheumatoid arthritis	ICAM1, IL6, TNF, IFNG, VEGFA, TGFB1, MMP1	7
HIF-1 signaling pathway	IL6, HIF1A, IFNG, EDN1, VEGFA, NOS3, STAT3	7
TNF signaling pathway	VCAM1, ICAM1, IL6, TNF, PTGS2, MMP9, EDN1	7
Jak-STAT signaling pathway	IL4, IL6, LEPR, IFNG, JAK3, IL10, STAT3	7
Cytokine-cytokine receptor interaction	IL4, IL6, TNF, LEPR, IFNG, TGFB1, IL10	7
African trypanosomiasis	VCAM1, ICAM1, IL6, TNF, IFNG, IL10	6
Leishmaniasis	IL4, TNF, PTGS2, IFNG, TGFB1, IL10	6

## Data Availability

The data used to support the findings of this study are included within the article and the supplementary information files.

## Supplementary Materials

Supplementary Materials Table S1 (sheet1): the active ingredients in SM and RP. Chemname was obtained from TCMSP. Table S1 (sheet2): corrected protein name. Entry was converted to gene name by UniProt. Table S1 (sheet3): SM and RP have common targets. Table S2: targets related to CCVDs. All targets from the TTD, NCBI gene, and OMIM database. Table S3: common targets of disease and drug. Table S4 (sheet1–sheet3): GO enrichment analysis: biological process (BP), cellular component (CC), and molecular function (MF). Table S4 (sheet4): KEGG pathway enrichment analysis. All data are from DAVID database. Supplementary Document 1: the structure of active ingredients data.
